# Supplementary material for: Transformation of a temporal speech cue to a spatial neural code in human auditory cortex
Source: eLife. 2020 Aug 25;9:e53051. doi: 10.7554/eLife.53051 (PMC7556862; doi:10.7554/eLife.53051)
Supplement: Supplementary file 1. — Each participant had ECoG grid coverage of one hemisphere (Hem), either left (LH) or right (RH). Participants completed as many trials as they felt comfortable with. Number of trials per participant for ECoG analyses indicate trials remaining after artifact rejection. Some participants chose to listen passively to some or all blocks, so three participants have no trials for behavioral analyses. See Materials and methods for description of inclusion criteria for individual trials in ECoG and behavioral analyses. A subset of speech-responsive (SR) electrodes on the lateral surface of the temporal lobe had a peak amplitude that was sensitive to VOT, selectively responding to either voiceless (V-) or voiced (V+) stimuli. See Materials and methods for details on electrode selection. [file elife-53051-supp1.docx]

| Participant | Hem | # trials  (ECoG) | # trials  (behavior) | # elecs  (SR) | # elecs  (VOT) | # elecs  (V- / V+) |
| --- | --- | --- | --- | --- | --- | --- |
| P1 | LH | 234 | 230 | 78 | 12 | 5 / 7 |
| P2 | RH | 625 | 592 | 56 | 8 | 6 / 2 |
| P3 | RH | 339 | 0 | 50 | 7 | 1 / 6 |
| P4 | LH | 333 | 0 | 40 | 7 | 3 / 4 |
| P5 | RH | 119 | 0 | 47 | 8 | 0 / 8 |
| P6 | RH | 305 | 277 | 36 | 5 | 0 / 5 |
| P7 | LH | 110 | 105 | 39 | 2 | 1 / 1 |
