# Supplementary material for: Transformation of a temporal speech cue to a spatial neural code in human auditory cortex
Source: eLife. 2020 Aug 25;9:e53051. doi: 10.7554/eLife.53051 (PMC7556862; doi:10.7554/eLife.53051)
Supplement: Supplementary file 2. — m = minimum activation level. Μ = maximum activation level. ρ = resting activation level. λ = decay rate. θ = propagation threshold. [file elife-53051-supp2.docx]

|  |  | **activation parameter** | | | | |
| --- | --- | --- | --- | --- | --- | --- |
|  |  | $\boldsymbol{m}$ | $\boldsymbol{M}$ | $\boldsymbol{\rho}$ | $\boldsymbol{\lambda}$ | $\boldsymbol{\theta}$ |
| **model node** | **Burst** | -10 | 10 | 0 | 1 | 0 |
|  | **Voicing** | -10 | 10 | 0 | 1 | 0 |
|  | **Inhibitor** | -10 | 10 | 0 | 1 | 0 |
|  | **Gap** | -10 | 10 | 0 | 0.25 | 0.25 |
|  | **Coincidence** | -10 | 10 | 0 | 0.25 | 1 |
