# Supplementary material for: Transformation of a temporal speech cue to a spatial neural code in human auditory cortex
Source: eLife. 2020 Aug 25;9:e53051. doi: 10.7554/eLife.53051 (PMC7556862; doi:10.7554/eLife.53051)
Supplement: Supplementary file 3. — The table is sparse, meaning that inputs to both Burst and Voicing detector units are 0 whenever a cell is blank. Inputs are clamped onto either Burst or Voicing detector units (always with strength = 1) for a given simulated VOT stimulus during the cycles that are labeled with a B or a V. [file elife-53051-supp3.docx]

| VOT | 0 |  |  | BV |  |  |  |  |  |  |  |  |  |  |  |  |
| --- | --- | --- | --- | --- | --- | --- | --- | --- | --- | --- | --- | --- | --- | --- | --- | --- |
|  | 10 |  |  | B | V |  |  |  |  |  |  |  |  |  |  |  |
|  | 20 |  |  | B |  | V |  |  |  |  |  |  |  |  |  |  |
|  | 30 |  |  | B |  |  | V |  |  |  |  |  |  |  |  |  |
|  | 40 |  |  | B |  |  |  | V |  |  |  |  |  |  |  |  |
|  | 50 |  |  | B |  |  |  |  | V |  |  |  |  |  |  |  |
|  |  | -2 | -1 | 0 | 1 | 2 | 3 | 4 | 5 | 6 | 7 | 8 | 9 | 10 | 11 | … |
|  |  | time post onset (cycles) | | | | | | | | | | | | | | |
